# *CD274* Knockout Promotes Bone Formation and Reduces Osteoclast Activity

**DOI:** 10.1007/s00223-026-01627-2

**Published:** 2026-10-20

**Authors:** Hadi El Assaad, Marina Komrakova, Stefan Lienenklaus, Sebastian Decker, Claudia Neunaber, Katrin Bundkirchen

**Affiliations:** 1 Department of Trauma Surgery, Hannover Medical School, Carl-Neuberg-Straße 1, 30625 Hannover, Germany; 2 Department of Orthopedics, Trauma Surgery and Plastic Surgery, Leipzig University Hospital, Liebigstraße 20, 04103 Leipzig, Germany; 3 Department of Trauma Surgery, Orthopaedics and Plastic Surgery, Goettingen University Medical Center, Robert-Koch-Str. 40, 37075 Goettingen, Germany; 4 Institute of Laboratory Animal Science, Hannover Medical School, Carl-Neuberg-Straße 1, 30625 Hannover, Germany

**Keywords:** Osteoporosis, In-vivo, PD-L1, Programmed death ligand 1, CD274, Knockout mice

## Abstract

Osteoporosis is a systemic disease characterized by decreased bone density, and deterioration in the micro-architectural remodelling of bone tissue, leading to an increased susceptibility to bone fractures. One molecule implicated in bone metabolism is Programmed Death Ligand 1 (PD-L1), a pharmacologically targetable immune-checkpoint molecule encoded by the gene Cd274. The aim of this study was to investigate the bone phenotype of Cd274 knockout (KO) mice at 26 weeks of age compared to C57BL/6J control wildtype mice on radiological, histological and biomechanical level. The cortical wall thickness and cortical volume increased, and the trabecular space decreased significantly in both femur and tibia of Cd274 KO mice. Moreover, there was a significant increase in BV/TV and trabecular volume of lumbar vertebrae of Cd274 KO mice. Yield load, stiffness, anorganic amount increased and organic amount decreased significantly in femoral bones of Cd274 KO mice. Additionally, there was a significantly higher number of osteocytes and lower number of osteoclasts in both humerus and lumbar vertebra of Cd274 KO mice. The findings demonstrate that constitutive knockout of Cd274 was associated with significantly increased bone mass and strength. These results—obtained in healthy, skeletally mature constitutive knockout mice—indicate an improved bone phenotype in the absence of Cd274 and thus contrast with previous studies that associate disruption of the PD-1/PD-L1 axis, including immune checkpoint inhibitor therapy, with bone loss and increased fracture risk. Further studies, particularly in dedicated osteoporosis models, are required to elucidate the influence of CD274 on bone metabolism before translational conclusions can be drawn.

**Supplementary Information:**

The online version contains supplementary material available at https://doi.org/10.1007/s00223-026-01627-2.

## Introduction

Osteoporosis is a systemic disease characterized by decreased bone density, as well as a deterioration in the micro-architectural remodeling of bone tissue, leading to an increased susceptibility to fractures [1]. At the pathophysiological level, the disease develops when bone resorption exceeds bone formation, resulting in progressive loss of skeletal mass. This imbalance is accentuated with advancing age as peak bone mass is reached during the third decade [2]. Consequently, bone trabeculae become thinner, osteon size decreases, and Haversian canals and marrow spaces enlarge, ultimately reducing bone strength and increasing the risk of fractures [3]. Osteoporotic fractures heal in a slower fashion, making them associated with impaired mobility, reduced quality of life, and increased mortality [4, 5].

Beside aging, several risk factors contribute to the emergence of osteoporosis, including postmenopausal women, use of corticosteroids, low body mass index, smoking, physical inactivity, alcohol intake and vitamin D deficiency [6, 7]. Some therapies such as bisphosphonates, selective estrogen-receptor modulators, denosumab and teriparatide could result in severe side effects, lose their effectiveness with long-term use or are even ineffective in some patients. Consequently, the development of new medications to treat osteoporosis is of high interest [8].

In a previous study of our group, it was demonstrated that human bone marrow stromal cells (hBMSC) from osteoporotic patients expressed significantly lower levels of programmed death ligand 1 (PD-L1) compared to healthy counterparts, while the expression of other surface antigens remained unchanged [9]. This finding highlights PD-L1 as a protein of particular interest for osteoporosis research and potential therapeutic future approaches. PD-L1 is a 290-amino-acid type I transmembrane protein encoded by the *CD274* gene, located on human chromosome 9 and mouse chromosome 19. It was the first ligand functionally characterized for the co-inhibitory programmed death receptor 1 (PD-1) and its expression is primarily regulated by toll-like receptors. Through binding to PD-1, PD-L1 contributes to the maintenance of peripheral and central immune tolerance [10, 11], thereby protecting tissues from immune-mediated damage [12]. Constitutive expression has been reported in T and B cells, dendritic cells, macrophages, mesenchymal stem cells, and bone marrow-derived mast cells [13]. Furthermore, PD-L1 is also found on non-hematopoietic cells such as microvascular endothelial cells, where it can be upregulated in response to certain interferons, including type I and II [14, 15]. Taken together, these observations suggest that *CD274* may be involved in the regulation of bone metabolism and could exert a protective role in the pathogenesis of osteoporosis.

Immune checkpoint inhibitors (ICIs) are drugs targeting different immune checkpoints which regulate immune responses to prevent tissue damage and autoimmunity. Cancer cells exploit immune checkpoints to evade immune destruction, making checkpoint inhibition a strategy to overcome tumor immune evasion. PD-1 inhibitors (Nivolumab, Pembrolizumab, Cemiplimab), PD-L1 inhibitors (Atezolizumab, Durvalumab, Avelumab) are some of the FDA approved ICIs [16]. Therefore, if silencing or blocking CD274/PD-L1 proves beneficial for bone mass and strength, the clinically approved PD-L1 inhibitors could, in principle, be repurposed or monitored for effects on bone health in patients.

In consequence, the aim of this study was to investigate the bone phenotype of *Cd274* knockout (KO) mice at 26–34 weeks of age compared to control mice on radiological, histological and biomechanical levels. The main hypothesis was that the *Cd274* KO mice show a decrease in bone mass compared to control mice.

## Methods

### Experimental Animals

26 weeks old B7-H1-/- *Cd274* KO mice (sacrificed for another scientific project and kindly provided by Prof. Schirmbeck) and 26–34 weeks old C57BL/6J control mice (purchased from Janvier Labs, Le Genest-Saint-Isle, France) were housed under standardized conditions in the central animal facility of the University Hospital Ulm or the Hannover Medical School (MHH). Animals were kept under standardized temperature (21 °C ± 2 °C), relative humidity (50%), and artificial light-dark cycles (14 h light, 10 h dark). They were fed ad libitum (Altromin 1324 TPF; Altromin GmbH, Lage, Germany) with free access to water. For sacrifice, the animals were anesthetized with isoflurane and cervical dislocation was performed. The bones (femora, humeri, tibiae, lumbar vertebrae L5) from eleven B7- H1-/- *Cd274* KO mice (two female, nine male) and from eleven wildtype C57BL/6J mice (three female, eight male), hereafter referred to as the control group (Ctrl), were carefully dissected and the surrounding soft tissue was removed. Collected bone samples were fixed for 24–48 h in 4% buffered formalin and transferred to 70% ethanol for long term storage until samples were procured to different analysis (see Table 1).

**Table 1:** Bone samples and performed analysis

Bone	Analysis
Femora	Micro-CT (right), Histology (left), Biomechanics (right and left), Ashing (right and left);
Tibiae	Micro-CT (right)
Humeri	Histology (left)
Vertebrae	Micro-CT, Histology

### Micro-CT

**Fig. 1:**
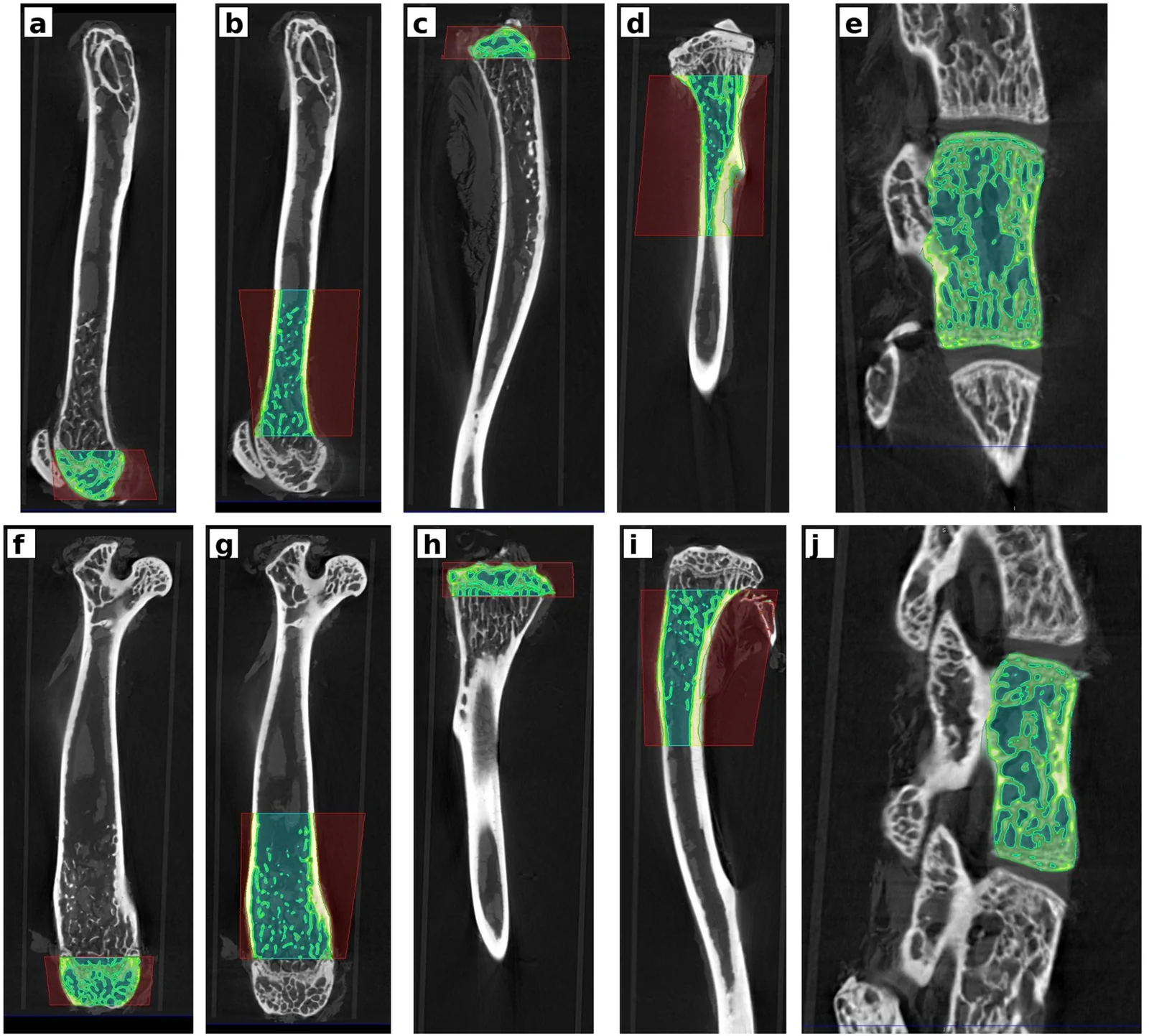
Sagittal and frontal micro-CT images showing the regions of interest used for morphometric analysis: **a**, **f** femur, epiphyseal region; **b**, **g** femur, diaphyseal region; **c**, **h** tibia, epiphyseal region; **d**, **i** tibia, diaphyseal region; **e**, **j** lumbar vertebral body

Right femur, right tibia and lumbar vertebra 5 (L5) of five *Cd274* KO mice and six Ctrl mice were examined using micro-CT (Siemens Inveon µCT, Siemens AG, Munich, Germany) with the following settings as described before [17]: 360°, 360 steps, 7 s Integration/Position with 55 kV, 500µA, Filter 1 (0.8 carbon) and 8.17 μm effective pixel size. Data were analyzed using the software Inveon Research Workplace, Version 4.2 (Siemens AG). A specific threshold of grey values and a set region of interest was determined and used for all evaluations to calculate total bone volume (mm^3^), cortical volume (mm^3^), cortical wall thickness (mm), trabecular spacing (mm) and the bone volume fraction (bone volume/total volume, BV/TV) in the distal epiphysis and diaphysis of the femur and tibia and in the whole vertebral body of L5. Regions of interest (ROIs) were defined separately for each skeletal site: a distal epiphyseal (trabecular) ROI and a mid-diaphyseal (cortical) ROI for the femur and tibia, and the entire vertebral body for L5. Contours were drawn manually on every fifth transverse slice and interpolated across intervening slices. A detailed, step-by-step ROI protocol is provided in Supplementary Methods S1 (Online Resource 1). Representative micro-CT images of the analysed regions of interest are provided in Fig. 1.

### Biomechanical Testing

Biomechanical parameters of the right and left femora from six *Cd274* KO mice and five control mice were assessed using a Zwick testing machine (145,660 Z020/TND; Zwick GmbH & Co. KG, Ulm, Germany) [18–20]. Both ends of femurs were positioned on an aluminum support base in such a way that they could bend but were prevented from tilting laterally, as shown in Fig. 4. The femur was loaded at the mid-diaphysis using a centrally positioned loading stamp, at a constant displacement rate of 5 mm/min with an initial preload of 1 N.

Following this fixation and positioning protocol, measurements were performed with a precision of 0.2–0.4% in the force range between 2 N and 500 N, and data were recorded using the testXpert software (Zwick GmbH & Co. KG, Ulm, Germany) [21].

The reported parameters included the yield load (N), end point of elastic deformation of bone defined as a decrease of the curve more than one standard deviation, and the stiffness (N/mm), the slope of the linear portion of the force–displacement curve. Both parameters were calculated using Microsoft Excel (MS Office 2016, Microsoft Corporation, Redmond, WA) [21].

### Ashing

To assess the organic and anorganic components of the femoral bone, five femora of *Cd274* KO mice and five femora of Ctrl mice previously used in biomechanical testing were subjected to the ashing process at 750 °C for one hour. The anorganic and organic masses were determined as percentages of the initial wet mass of each femur, measured before ashing [22].

### Histology

#### Stainings

Left femur, left humerus and lumbar vertebra 5 of five *Cd274* KO mice and six Ctrl mice were decalcified in 20% EDTA solution, pH 7.4. Prior to decalcification, the bones were rinsed for 1 min in distilled water. Decalcification was carried out for 14 days at 4 °C under continuous agitation at a bone-to-solution ratio of 1:20 (approximately 15 ml EDTA solution per mouse bone). The solution was changed two to three times per week and decalcification was considered complete when the bone was soft and pliable. The samples were then rinsed three times for 1 min in distilled water, dehydrated in an ascending ethanol series (70% ethanol for at least 1 h, 90% for 45 min and 100% for 45 min, followed by three 1-h changes of 100%), cleared in xylene (two 45-min changes followed by 1 h), infiltrated with paraffin (three 30-min changes followed by 1 h) and embedded in paraffin. All samples were cut into 5 μm thick sections using a microtome (Reichert Jung AutoCut 2040, Cambridge Instruments GmbH, Nußloch, Germany). Before staining, all sections were deparaffinised in xylene and rehydrated through a descending alcohol series. To analyze the amount of cartilage, as well as the number of osteocytes, sections were stained with Light Green/Safranin O (10 min in Iron Hematoxylin [Carl Roth GmbH, Karlsruhe, Germany], 5 min in Light Green [Sigma-Aldrich Chemie GmbH, Munich, Germany], 5 min in 1% Safranin O [Merck, Darmstadt, Germany]), as previously described [23]. Representative staining is shown in Supplementary Figure S1.

For counting the osteoclasts [24] staining with Tartrate-resistant acid phosphatase (TRAP) was performed 20 min in acetate buffer [J.T.Baker, Deventer, Netherlands], 2 min in TRAP staining solution containing Naphthol AS-MX Phosphate [No. N4875, Sigma-Aldrich, Steinheim, Germany] and Fast Red TR Salt [No. 368881-25G Sigma-Aldrich], 1 min in Mayer’s hemalum [Merck]). Representative TRAP staining is shown in Supplementary Figure S2. For evaluating mineralized bone, pentachrome staining (10 min in alcian blue [Sigma-Aldrich, Steinheim, Germany], 60 min in alkaline ethanol, 10 min in Weigert’s iron hematoxylin [Carl Roth, Karlsruhe, Germany], 15 min in Brilliant Croceine R-Acid Fuchsine O [Chroma/Waldeck, Münster, Germany], 20 min in phosphotungstic acid, and 60 min in safran du Gâtinais) was employed to differentiate the bone matrix and surrounding tissues, as described by Bundkirchen et al. [25]. Representative pentachrome staining is shown in Supplementary Figure S3.

#### Evaluation

Digital images of bone sections were acquired using VHX digital microscope 7000 (Keyence GmbH, Germany). Light Green/Safranin O-stained sections were analyzed at 300x magnification to assess cartilage surface and to count osteocytes (Supplementary Figure S1). The amount of cartilage was quantified as the Safranin O–positive (red) area expressed as a percentage of the total epiphyseal area in the distal femur and proximal humerus (Supplementary Figure S1b). Similarly, TRAP and Pentachrome-stained sections were examined at the same magnification for cell quantification and mineralized bone evaluation (Supplementary Figures S2 and S3) respectively. All histological quantification was performed by a single assessor blinded to genotype. One section per bone was evaluated for each stain.

For the evaluation of mineralized bone, expressed as a percentage of the total bone area in the distal femur and proximal humerus (diaphysis and epiphysis) as well as in vertebral bodies, and cartilage in distal femur and proximal humerus, a self-written Java software named “Image Filter Tool”, based on the openCV library (version 4.1.0), was used.

Osteoclasts were counted in a set region of interest in the diaphysis of the femur and humerus (one ROI, 1.55 mm^2^ and 0.66 mm^2^ respectively) and in vertebral bodies (three ROIs, 0.04 mm^2^ each) in TRAP-stained slices (ROIs are illustrated in Supplementary Figure S2). Osteocytes were counted in the cortex of the femur and humerus (two ROIs, 0.06 mm^2^ each, illustrated in Supplementary Figure S1a, c)) and in vertebral bodies (three ROIs, 0.04 mm^2^) in Light Green/Safranin O-stained slices, as previously described [23]. Oc.S/BS was quantified in TRAP-stained slices using TrapHisto, an open-source software for semi-automated histomorphometry of bone resorption parameters [26].

Marrow adipocytes were quantified on the Light Green/Safranin O-stained sections, in the osteoclast regions of interest of the femur and humerus and in all marrow of one 300x field of the L5 vertebral body (Supplementary Figure S1a). Since lipid is extracted during paraffin processing, adipocytes were identified as round to oval, optically empty profiles with a thin membrane, surrounded by haematopoietic marrow. Vessel lumina, retraction clefts and empty lacunae were not counted. Candidates were detected with a custom Python script and confirmed visually. Adipocyte number and area per marrow area (N.Ad/Ma.Ar, Ad.Ar/Ma.Ar) and the mean adipocyte diameter (Ad.Dm) were calculated according to the nomenclature of the International Bone Marrow Adiposity Society [27]; the detailed protocol is provided in Supplementary Methods S2 (Online Resource 1).

Control and *Cd274* KO sections of all stainings are shown side by side in Supplementary Figure S5.

### Statistical Analysis

Statistical analysis was performed using GraphPad Prism 9.0. Distribution normality was confirmed by the Anderson-Darling test and by interpreting the distribution plots. Analysis between the *Cd274* KO mice and controls were performed using unpaired t-test. Data are presented in the figures as scatter dot plots and reported throughout the Results as mean values with 95% confidence interval (CI). The significance level was set at *p* ≤ 0.05.

## Results

### Enhanced Cortical Wall Thickness, Cortical Volume and Decreased Trabecular Space in Both Femur and Tibia of *Cd274* KO Mice

**Fig. 2:**
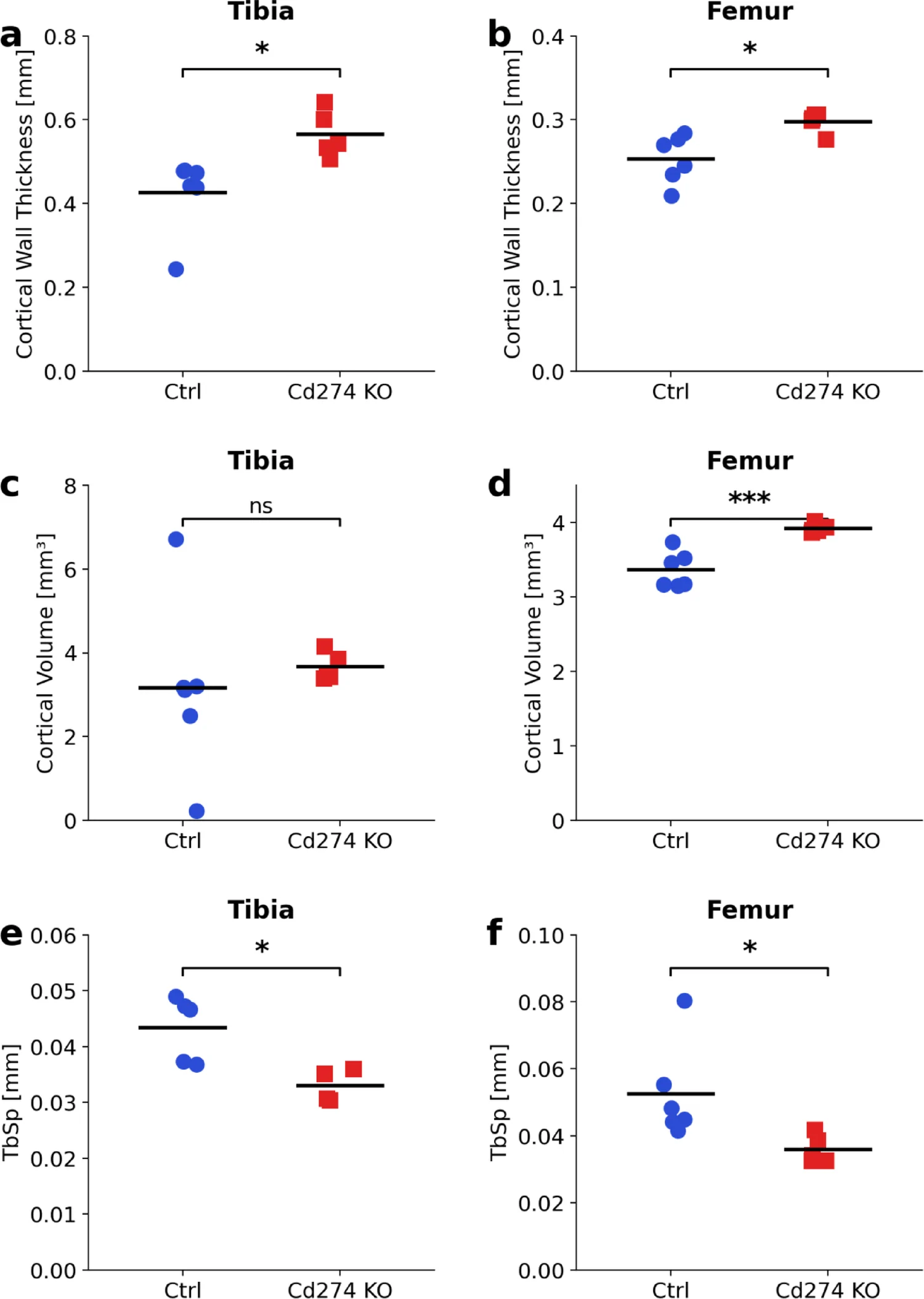
Bone morphometry of tibia and femur. Cortical wall thickness of tibial (**a**) and femoral (**b**) diaphyses, cortical volume of tibial (**c**) and femoral (**d**) diaphyses, trabecular space of tibial (**e**) and femoral (**f**) epiphyses in control and *Cd274* KO mice (*n* = 5–6/group for tibia, *n* = 5–6/group for femur). *p-value < 0.05, ***p-value < 0.001, TbSp: Trabecular Space, ns: non-significant

A knockout of *Cd274* led to a significantly higher cortical wall thickness in tibial diaphysis (0.565 mm; 0.496–0.633 mm) compared to the control group (0.426 mm; 0.331–0.521 mm; *p* = 0.016; Fig. 2a). Similarly, the cortical wall thickness of femoral diaphysis was significantly increased in *Cd274* KO mice (0.298 mm; 0.282–0.313 mm) in comparison to the control group (0.253 mm; 0.223–0.284 mm; *p* = 0.011; Fig. 2b).

The cortical volume of the tibial diaphysis did not differ significantly between the two groups (*p* = 0.608, Fig. 2c). In contrast, the cortical volume of the femur diaphysis was significantly higher in *Cd274* KO mice (3.920 mm³; 3.852–3.995 mm³) compared to the control group (3.370 mm³; 3.114–3.626 mm³; *p* < 0.001; Fig. 2d).

The trabecular space (TbSp) in the epiphysis of the tibia was significantly decreased in *Cd274* KO mice (0.033 mm; 0.028–0.037 mm) compared to the control group (0.043 mm; 0.036–0.051 mm; *p* = 0.015; Fig. 2e). Similarly, the trabecular space in the epiphysis of the femur was significantly lower in *Cd274* KO mice (0.036 mm; 0.031–0.041 mm) compared to the control group (0.052 mm; 0.037–0.067 mm; *p* = 0.037; Fig. 2f).

Trabecular number and trabecular thickness in the tibial and femoral epiphyses did not differ significantly between *Cd274* KO mice and the control group (Supplementary Figure S4). Furthermore, total bone volume in tibia and femur and lumbar vertebra was not significantly different between *Cd274* KO mice when compared to the control group (data not shown).

### Enhanced BV/TV and Trabecular Volume in Lumbar Vertebra of *Cd274* KO Mice

**Fig. 3:**
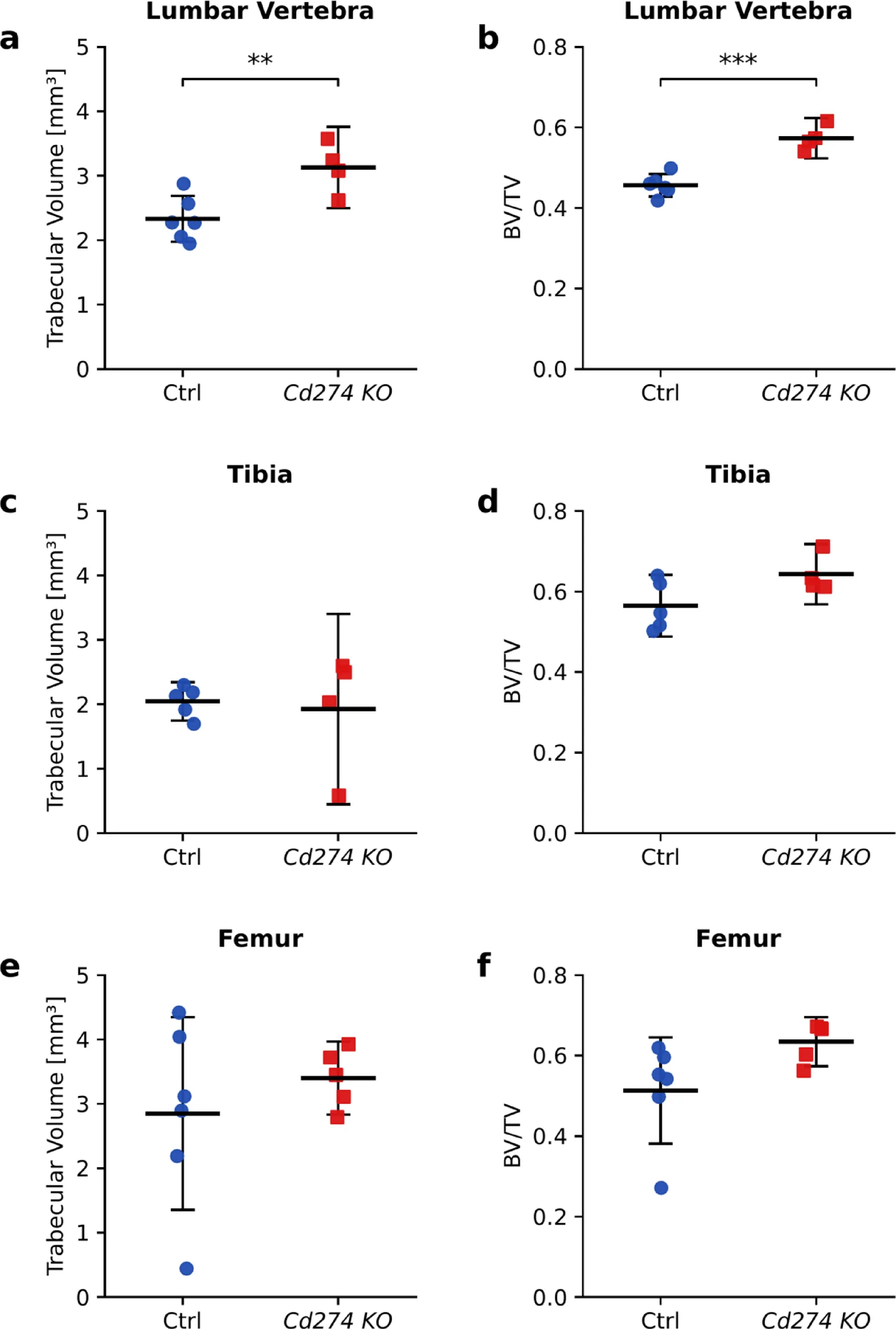
Bone volume parameters of lumbar vertebra, tibia and femur. Trabecular volume (mm³) and BV/TV of the lumbar vertebra (**a**, **b**), tibia (**c**, **d**) and femur (**e**, **f**) in control and *Cd274* KO mice (*n* = 4–6/group). **p-value < 0.01; ***p-value < 0.001. BV/TV: Bone volume fraction

In *Cd274* KO mice, the trabecular volume in the lumbar vertebra was significantly increased (3.126 mm³; 2.495–3.757 mm³) compared to the control group (2.331 mm³; 1.972–2.69 mm³; *p* = 0.009; Fig. 3a). Similarly, the BV/TV ratio was significantly higher in *Cd274* KO mice (0.573; 0.523–0.623) than in the control group (0.456; 0.428–0.484; *p* < 0.001; Fig. 3b). BV/TV and trabecular volume in tibia and femur did not differ significantly between *Cd274* KO group and the control group (Fig. 3c-f).

### Enhanced Yield Load, Stiffness, Anorganic Amount and Decreased Organic Amount in Femoral Bones of *Cd274* KO Mice

**Fig. 4:**
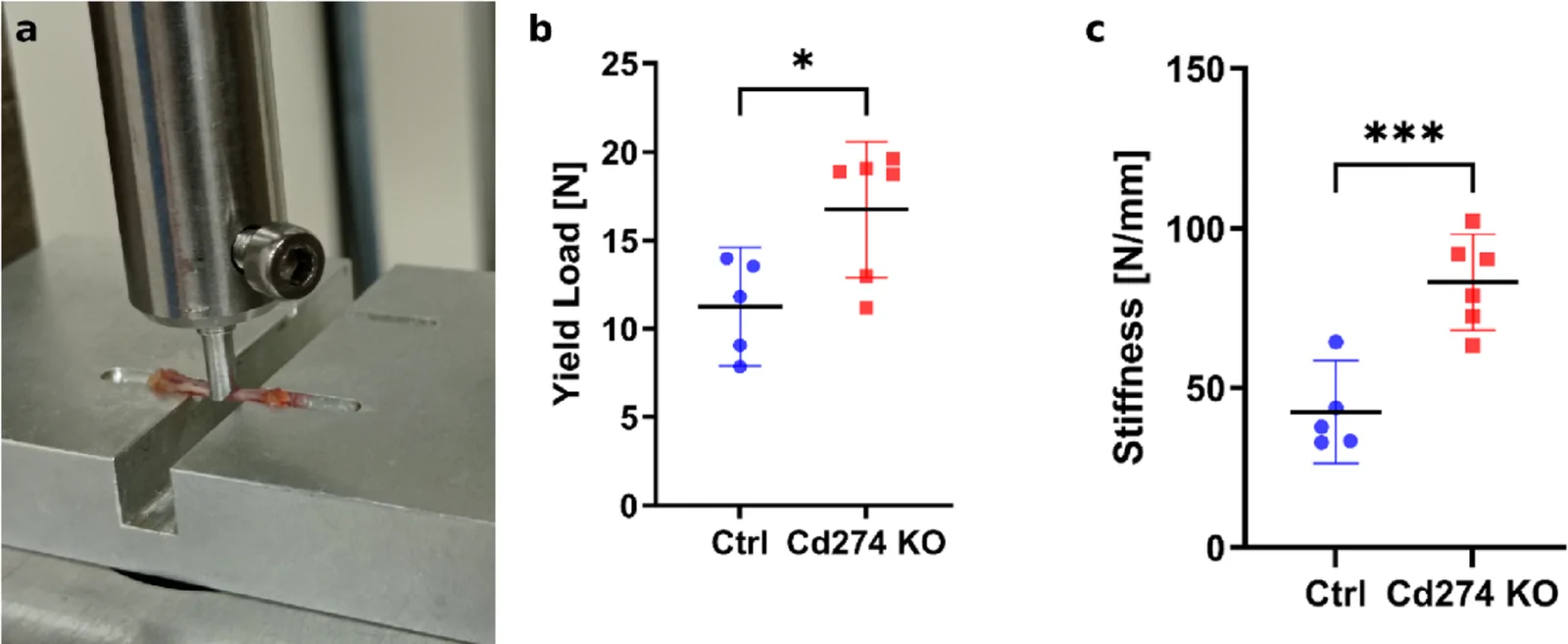
Biomechanical properties of femur. **a** Three-point bending test setup showing biomechanical testing of a right femur. Yield load (**b**) and stiffness **c** of the femur in control and *Cd274* KO mice (*n* = 5–6/group). *p-value < 0.05; ***p-value < 0.001

Knocking out *Cd274*, significantly increased yield load in the femoral bones (18.32 N; 15.13–21.21) compared to the control group (12.17 N; 10.42–13.93 N; *p* = 0.0016; Fig. 4b). Stiffness was also significantly increased in *Cd274* KO mice (82.41 N/mm; 10.09–94.73 N/mm) compared to the control group (51.37 N/mm; 40.14–62.6 N/mm; *p* = 0.0006; Fig. 4c).

**Fig. 5:**
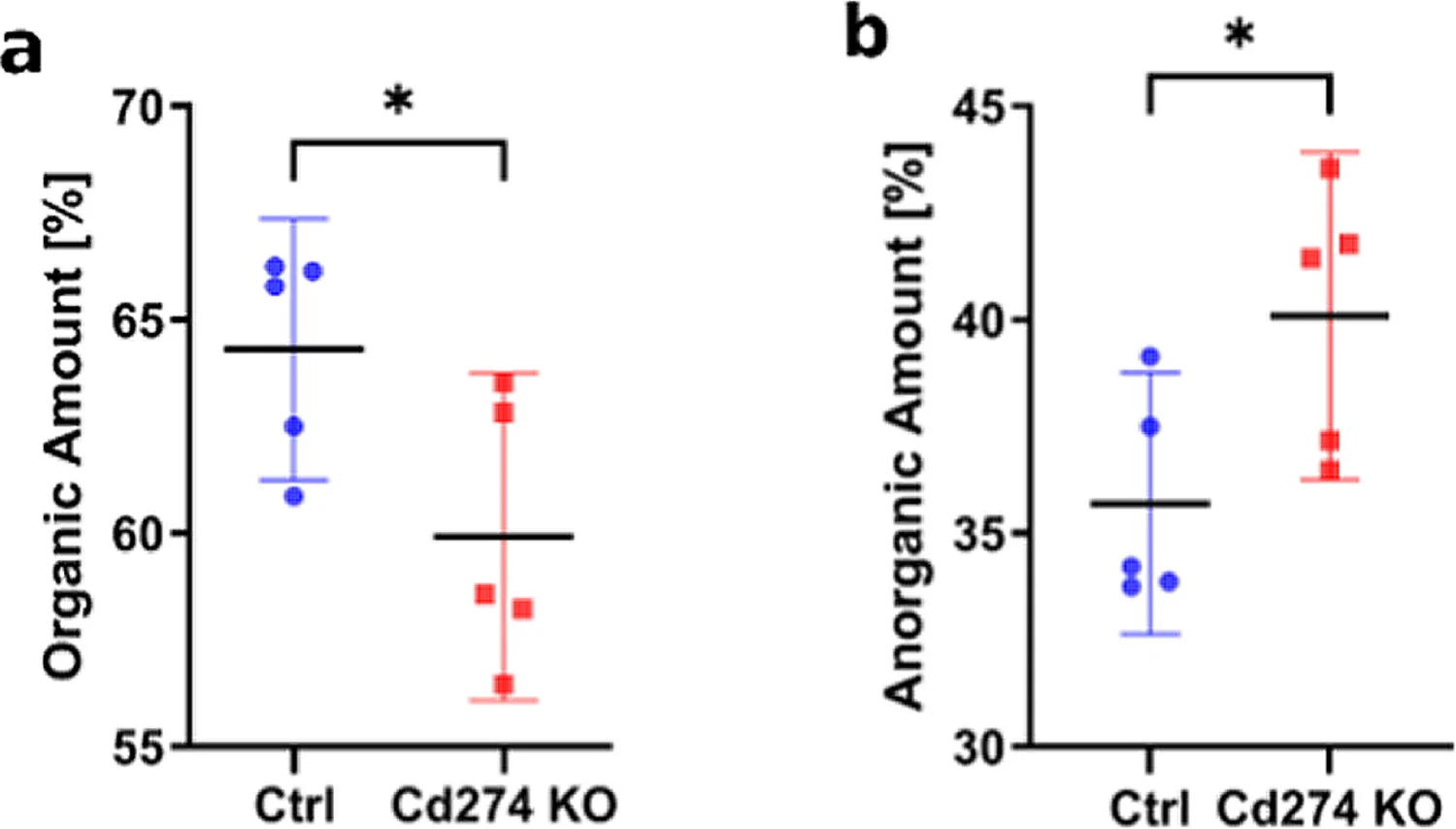
Composition of femur. Organic amount (**a**) and Anorganic amount (**b**) of femur in control and *Cd274* KO mice (*n* = 5/group). *p-value < 0.05

In terms of composition, anorganic amount of the femur was significantly higher in *Cd274* KO mice (40.08%; 36.25–43.91%) compared to the control group (35.69%; 32.63–38.76%; *p* = 0.038; Fig. 5a). Conversely, *Cd274* KO mice exhibited a significantly lower organic amount of the femoral bone (59.92%; 56.0–63.75%) compared to the control group (64.31%; 61.24–67.37%; *p* = 0.038; Fig. 5b).

### Histological Evaluation

**Fig. 6:**
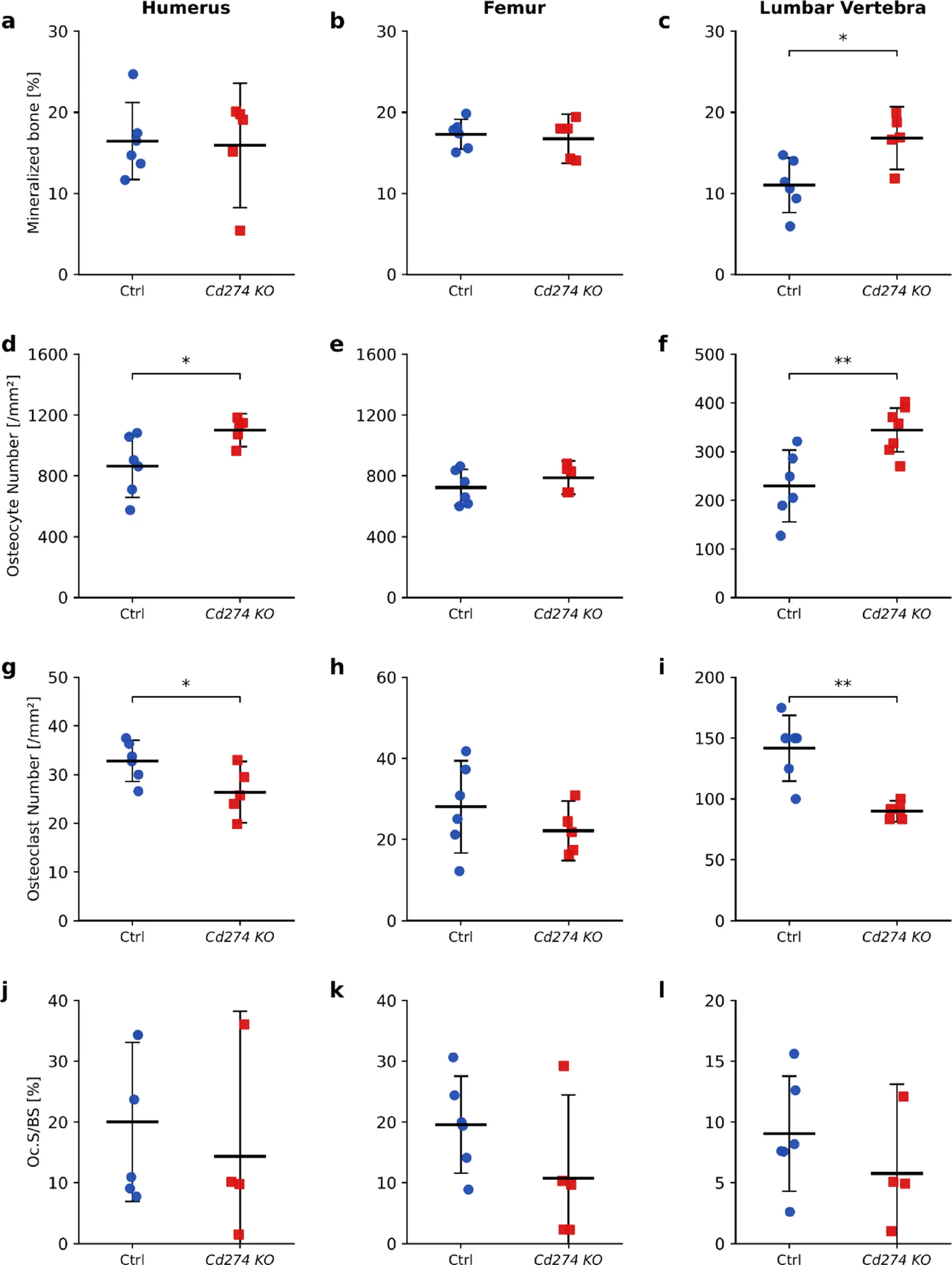
Histological analysis in femur, humerus and lumbar vertebra. Mineralized bone (in %) in humerus (**a**), femur (**b**) and vertebrae (**c**), osteocyte number per mm² in humerus (**d**), femur (**e**) and lumbar vertebra (**f**), osteoclast number per mm² in humerus (**g**), femur (**h**) and lumbar vertebra (**i**), and osteoclast surface per bone surface (Oc.S/BS, %) in humerus (**j**), femur (**k**) and lumbar vertebra (**l**) in control and *Cd274* KO mice (*n* = 5–6/group). *p-value < 0.05; **p-value < 0.01

Mineralized bone% of the *Cd274* KO mice in the humerus (15.9%; 8.23–23.58%) and femur (16.75%; 13.71–19.79%) showed no significant differences compared to their respective control animals (humerus: 16.45%; 11.69–21.21%; *p* = 0.871; Fig. 6a; femur: 17.31%; 15.46–19.15%; *p* = 0.672; Fig. 6b). However, mineralized bone in the lumbar vertebrae was significantly higher in *Cd274* KO mice (16.82%; 12.96–20.67%) compared to the control group (11.01%; 7.643–14.38%; *p* = 0.014; Fig. 6c; Supplementary Figure S5u, v).

The number of osteocytes was significantly higher in the humerus (1102/mm²; 994.3–1209/mm²) and lumbar vertebra (340.9/mm²; 281.9–399.9/mm²) of *Cd274* KO mice compared to their respective controls (humerus: 868.8/mm²; 659.1–1072/mm²; *p* = 0.036; Fig. 6d; Supplementary Figure S5g, h; lumbar vertebra: 225.2/mm²; 155.6–294.9/mm²; *p* < 0.01; Fig. 6f; Supplementary Figure S5i, j). Osteocyte numbers in the femur did not differ between the groups (*Cd274* KO: 788.5/mm²; 678.7–898.3/mm²; Ctrl: 723.4/mm²; 604.4–842.3/mm²; *p* = 0.324; Fig. 6e).

The number of osteoclasts was significantly lower in the humerus (26.4/mm²;20.1–32.69/mm²) and lumbar vertebra (90/mm²; 81.34–98.66/mm²) of *Cd274* KO mice compared to their respective control groups (humerus: 32.83/mm²; 28.58–37.08/mm²; *p* = 0.044; Fig. 6g; Supplementary Figure S5m, n; lumbar vertebra: 141.7/mm²; 114.6–168.8/mm²; *p* = 0.002; Fig. 6i; Supplementary Figure S5o, p). The osteoclast number in the femur was also lower in KO mice (22.13/mm²; 14.82–29.45/mm²) but did not reach statistical significance compared to the control group (28.07/mm²; 16.69–39.46/mm²; *p* = 0.304; Fig. 6h, Supplementary Figure S2). Osteoclast surface per bone surface (Oc.S/BS) was lower in *Cd274* KO mice at all three sites, but the differences did not reach statistical significance (femur: median 9.68%; IQR 2.32–10.31% vs. 19.66%; 15.44–23.29%; *p* = 0.178; humerus: 9.98%; 7.73–16.63% vs. 17.32%; 9.55–31.67%; *p* = 0.762; lumbar vertebra: 5.00%; 3.95–6.82 vs. 7.90%; 7.58–11.51%; *p* = 0.257). The Oc.S/BS values for all three skeletal sites are shown in Fig. 6j–l.

The cartilage percentage showed no statistical difference between the control and KO mice in the distal femur and proximal humerus (data not shown).

The adipocyte number per marrow area did not differ significantly between Cd274 KO mice and the control group in the femur (14.87/mm^2^; 11.23–19.94/mm^2^ vs. 24.44/mm^2^; 8.61–28.39/mm^2^; *p* = 0.537), the humerus (10.57/mm^2^; 10.32–17.99/mm^2^ vs. 1.68/mm^2^; 1.57–6.59/mm^2^; *p* = 0.247) or the lumbar vertebra (0/mm^2^; 0–3.32/mm^2^ vs. 1.37/mm^2^; 0–2.95/mm^2^; *p* = 0.914). The same applied to the adipocyte area per marrow area (femur 0.37% vs. 1.21%, *p* = 0.429; humerus 0.44% vs. 0.03%, *p* = 0.247) and to the mean adipocyte diameter, which was lower in the femur of *Cd274* KO mice without reaching significance (19.80 μm; 18.50–21.60 μm vs. 23.85 μm; 23.40–24.75 μm; *p* = 0.052; data not shown).

## Discussion

Osteoporosis is a common disease. Epidemiologically, around 9 million fractures worldwide result from osteoporosis every year, where this disease affects more females than males, especially in older ages and in the post-menopausal stage [28, 29]. One molecule implicated in the regulation of bone metabolism is PD-L1, which is encoded by the gene *CD274*. In a previous study, our group detected a lower expression of *CD274* in BMSCs from osteoporotic patients [9]. Therefore, the hypothesis for this study was that *Cd274* KO mice would show lower bone mass than control animals. Consequently, the bone phenotype of *Cd274* KO mice was compared to control mice radiologically, biomechanically, biochemically and histologically. However, the assumed hypothesis was not confirmed, as the analysis showed contradictory results. Contrary to expectations, constitutive knockout of *Cd274* was associated with significantly increased bone mass and strength.

Our results revealed that *Cd274* KO mice aged 26 weeks, exhibited significantly higher femoral cortical volume. This may suggest enhanced bone formation or reduced bone resorption in *Cd274* KO mice. However, this finding is conflicted with results by Greisen et al. where bone area and cortical thickness was decreased in PD-L1 KO mice of male sex aged 8 weeks [30], and in another study by Schildberg et al., where PD-1 and PD-L1 KO mice showed reduced bone density compared to controls [31]. This discrepancy may relate to differences in the age, sex and developmental stage of the animals. Greisen et al. examined skeletally immature male mice at 8 weeks of age [30], while Schildberg et al. did not specify the age or sex of the animals studied [31]. The present study instead analysed skeletally mature mice aged 26 weeks (CD274 KO) and 26–34 weeks (Ctrl), of mixed sex. As bone is still accruing rapidly in young animals, the skeletal consequences of *Cd274* loss may differ markedly between immature and mature mice. As *Cd274* is deleted constitutively, the phenotype observed at 26 weeks reflects the cumulative result of skeletal growth, modelling and remodelling in the lifelong absence of PD-L1, and the present design cannot separate an effect on skeletal development from an effect on remodelling in the mature skeleton. A developmental contribution is plausible: the reduced cortical and trabecular bone reported in 8-week-old PD-L1 knockout mice [30] and the increased cortical volume and reduced trabecular spacing observed here at 26 weeks would be compatible with altered bone accrual during skeletal growth followed by continued accrual once longitudinal growth has ceased, rather than with a constant effect of PD-L1 on bone throughout life. Distinguishing developmental programming from ongoing regulation of remodelling in the adult skeleton will require inducible or cell-type-specific deletion of *Cd274* in skeletally mature animals, together with an age series spanning growth and maturity and measures of longitudinal bone growth. Apart from differences in the animals’ ages, the types of radiographic examinations and the bone regions analyzed also vary between the studies, which further complicates a direct comparison. Greisen et al. assessed the femur only, by dual-energy X-ray absorptiometry of the whole femur and micro-CT of the distal femoral metaphysis (trabecular bone) and the mid-diaphysis (cortical bone) [30], whereas our study assessed the femur, tibia and lumbar vertebrae.

Additionally, CD274 KO mice demonstrated significant reduction of femoral and tibial trabecular space. Nagahama et al. published similar findings, demonstrating increased trabecular bone volume, trabecular thickness and trabecular number along with decreased trabecular separation in PD-1 KO mice compared to the controls at 12 weeks of age [32]. However, this also conflicts with the results of Greisen et al. who stated a significant reduction of trabecular bone volume fraction and trabecular spacing in both PD-1 KO and PD-L1 KO mice aging 8 weeks compared with controls [30].

To the best of our knowledge, this study is the first to compare the biomechanical properties (yield load and stiffness) in control and *Cd274* KO mice. Aligning with increased cortical bone and decreased trabecular space, there was a significant increase in femoral biomechanical properties in KO mice compared to controls. These findings support enhanced bone strength and improved resistance to deformation under mechanical stress when *Cd274* is silenced [33, 34].

Furthermore, KO mice exhibited significantly lower organic content and a higher anorganic content in femoral bone compared to controls while osteoporotic bone is characterized by reduced anorganic and increased organic components [6]. This is consistent with the increased biomechanical stiffness and micro-CT parameters observed and suggests a potential impact of *Cd274* deficiency on bone material composition and therefore lead to a potential reduction of fracture susceptibility. However, Greisen et al. showed significant lower areal bone mineral density of 8-week-old PD-L1 KO mice when compared with control mice [35].

The favourable effect of Cd274 KO on bone composition, structure and biomechanics could be explained by the decreased number of osteoclasts and increased number of osteocytes observed in the present study. The reduced osteoclast number suggests decreased bone resorption in these regions, which may contribute to the increased bone mass observed. One study showed results aligning with our study, where PD-1/PD-L1 inhibition impaired osteoclastogenesis [36]. In another study Nagahama et al. demonstrated a decreased ability of PD-1 KO mice’s hematopoietic precursor cells to differentiate into osteoclasts which may explain this reduction of osteoclasts [32]. In contrast, Schildberg et al. revealed an increased number of osteoclasts in the Cd274 KO mice [31].

The higher number of osteocytes in the humeri and lumbar vertebrae may be consistent with increased bone formation in PD-L1 KO mice. In contrast, other studies showed no effect of PD-1 silencing on osteogenesis [32, 36]. Gassner et al. demonstrated that neither the number of osteoblasts nor their activity was influenced by inhibition of PD1 and PD-L1 [36].

Concerning the immunomodulatory effect and bone homeostasis regulation, an in vitro study showed that differentiated osteoblasts express less proinflammatory cytokines which is mediated by the PD-1/PD-L1 axis [37]. Moreover, PD-L1 treatment increased the expression of osteogenic genes and thereby the osteogenic properties in osteoblasts [37]. In addition, an inhibition of the PD-1/PD-L1 axis reversed the anti-inflammatory effect, suggesting that disruption of the PD-1/PD-L1 signalling may contribute to inflammation-driven bone loss [37]. However, inflammatory parameters were not assessed in our study, but an increased bone mass rather than bone loss was observed.

Brom et al. showed that PD-1 and PD-L1 induction increased osteoclastogenesis, and the blockade using monoclonal antibodies resulted in decreased size and number [38]. However, human studies on ICIs showed discrepancy with our study’s results. In a cohort of 1600 ICI users (with a majority inhibiting PD-1), there was a two and a half times increase in fracture rate in the first year after ICI initiation as compared to the year prior to ICI initiation [39]. Moreover, another study enrolled a series of 44 patients affected by advanced non-small cell lung cancer or renal cell carcinoma, without bone metastases, and treated with PD-1 or PD-L1 as monotherapy. Results suggest a direct relationship between ICIs treatment and increased plasma levels of type I collagen C-terminal telopeptide (CTX), a marker of bone resorption. This reflects an increased osteoclast activity which can lead to an higher potential risk of fractures [40]. This was also seen in a case series, where serious vertebral fractures was reported in cancer patients lacking pre-existing risk factors and taking ICIs (PD-1 and PD-L1 inhibitors) [41]. However, these contradictory findings could be attributed to the effect of cancer itself on bone health rather than the ICIs [42]. Grassner et al., showed that in cancer patients without bone metastases, ICIs (PD-1 and PD-L1 inhibitors) transiently decreased CTX and later increased formation markers (procollagen type I N-terminal propeptide, osteocalcin). These results suggest a potential protective effect on bone rather than increased osteoporosis risk [36].

Marrow adipocytes were present in all three bones, but neither their number nor the marrow area they occupied differed between Cd274 KO mice and controls. This is consistent with the direction of the reported relationship between marrow fat and PD-L1: Costa et al. showed that an expansion of bone marrow adipose tissue increases the number of PD-L1 positive myeloid cells and thereby promotes osteoclastogenesis [43], placing marrow adiposity upstream of PD-L1. The present data suggest that the reverse does not apply, and that the higher bone mass in Cd274 KO mice is not accompanied by a shift of the marrow towards adipogenesis.

Despite careful planning, this study also has some limitations. The sample size for certain analyses may limit the generalizability of the findings. The availability of bone material was limited, precluding additional analyses such as RNA or protein expression studies. Therefore, the molecular mechanisms underlying the observed phenotypic changes were not investigated. Furthermore, the study was restricted to a single age group, preventing conclusions about potential age-related effects. As an age range of 26–34 weeks was used for the control group instead of an exact analysis time point of 26 weeks, which was used in the Cd274 KO group, a potential age-related influence on the observed differences cannot be excluded. Moreover, this study was neither designed nor powered to detect sex-specific effects. As sex can influence bone mass and microarchitecture, the use of animals of both sexes (KO: two female, nine male; Ctrl: three female, eight male) could be seen as a further limitation. Another restriction is the small group size, which is due to the limited availability of biological material from the knockout animals. For this reason, no a priori power analysis was conducted; instead, material from all knockout animals available at the time and a control group selected accordingly was analysed. While larger cohorts would increase statistical power and allow detection of more subtle effects, the present study was designed to detect robust, biologically meaningful differences, and the observed effects were consistent and of large magnitude. Importantly, this is a constitutive knockout in healthy adult mice and does not constitute an osteoporosis model, so the findings reflect lifelong Cd274 absence rather than therapeutic PD-L1 inhibition in compromised bone.

Building on these findings, several further analyses would help clarify the role of PD-L1 in bone. Future studies should explore the molecular pathways linking PD-L1 and bone metabolism and should examine both constitutive Cd274 loss and pharmacological PD-L1 inhibition across multiple ages and in established models of bone loss (e.g. ovariectomy-induced or age-related osteoporosis), incorporating dynamic histomorphometry and molecular analyses. Complementary histological read-outs, including osteocyte lacunar occupancy, together with circulating bone-turnover markers such as procollagen type I N-terminal propeptide (P1NP) and C-terminal telopeptide of type I collagen (CTX), would provide further insight into the underlying bone-remodelling processes and should be incorporated into future work. Larger cohorts would additionally increase statistical power and allow detection of more subtle effects.

Facing these contradictory results, the exact effect of PD-L1 silencing on bone health needs further investigation in the future. This article adds further information to the literature by showing findings from a PD-L1 KO model, differing from reports on ICI in osteoporosis.

## Conclusion

In conclusion, *Cd274* knockout mice exhibit significant alterations in bone morphology, mechanical properties and cellular composition, identifying *Cd274* as a modulator of the bone phenotype. These findings provide a foundation for further research into the molecular mechanisms governing bone–immune interactions. Their therapeutic relevance remains to be determined in dedicated osteoporosis models before any conclusion about PD-L1 inhibition for enhancing bone density or reducing fracture risk can be drawn.

## Supplementary Information

Below is the link to the electronic supplementary material.

Supplementary Material 1

## Data Availability

The data supporting the findings of this study are available upon reasonable request.

## References

[1] Glaser DL, Kaplan FS (1997) Osteoporosis: definition and clinical presentation. Spine 22:12S 10.1097/00007632-199712151-00003 9431639

[2] Varacallo MA, Fox EJ (2014) Osteoporosis and its complications. Med Clin N Am 98:817–831. 10.1016/j.mcna.2014.03.007. xii–xiii 24994054

[3] Varacallo MA, Seaman TJ, Jandu JS, Pizzutillo P (2025) Osteopenia. In: StatPearls. StatPearls Publishing, Treasure Island (FL) 29763053

[4] Roux C, Thomas T, Paccou J et al (2021) Refracture and mortality following hospitalization for severe osteoporotic fractures: the Fractos study. JBMR Plus 5:e10507. 10.1002/jbm4.10507 PMC8260818 34258503

[5] Morin SN, Leslie WD, Schousboe JT (2025) Osteoporosis: a review. JAMA 334:894–907. 10.1001/jama.2025.6003 40587168

[6] Porter JL, Varacallo MA (2025) Osteoporosis. In: StatPearls. StatPearls Publishing, Treasure Island (FL)

[7] Lips P, van Schoor NM (2011) The effect of vitamin D on bone and osteoporosis. Best Pract Res Clin Endocrinol Metab 25:585–591. 10.1016/j.beem.2011.05.002 21872800

[8] Deal C (2009) Potential new drug targets for osteoporosis. Nat Clin Pract Rheumatol 5:20–27. 10.1038/ncprheum0977 19098925

[9] Zeller A-N, Selle M, Gong Z et al (2021) Osteoporosis is accompanied by reduced CD274 expression in human bone marrow-derived mesenchymal stem cells. Eur Cell Mater 41:603–615. 10.22203/eCM.v041a39 34056703

[10] Boussiotis VA (2016) Molecular and biochemical aspects of the PD-1 checkpoint pathway. N Engl J Med 375:1767–1778. 10.1056/NEJMra1514296 PMC5575761 27806234

[11] Ritprajak P, Azuma M (2015) Intrinsic and extrinsic control of expression of the immunoregulatory molecule PD-L1 in epithelial cells and squamous cell carcinoma. Oral Oncol 51:221–228. 10.1016/j.oraloncology.2014.11.014 25500094

[12] Jin H-T, Ahmed R, Okazaki T (2011) Role of PD-1 in regulating T-cell immunity. Curr Top Microbiol Immunol 350:17–37. 10.1007/82_2010_116 21061197

[13] Yamazaki T, Akiba H, Iwai H et al (2002) Expression of programmed death 1 ligands by murine T cells and APC. J Immunol 169:5538–5545. 10.4049/jimmunol.169.10.5538 12421930

[14] Eppihimer MJ, Gunn J, Freeman GJ et al (2002) Expression and regulation of the PD-L1 immunoinhibitory molecule on microvascular endothelial cells. Microcirculation 9:133–145. 10.1038/sj/mn/7800123 PMC3740166 11932780

[15] Schreiner B, Mitsdoerffer M, Kieseier BC et al (2004) Interferon-beta enhances monocyte and dendritic cell expression of B7-H1 (PD-L1), a strong inhibitor of autologous T-cell activation: relevance for the immune modulatory effect in multiple sclerosis. J Neuroimmunol 155:172–182. 10.1016/j.jneuroim.2004.06.013 15342209

[16] Arafat Hossain M (2024) A comprehensive review of immune checkpoint inhibitors for cancer treatment. Int Immunopharmacol 143:113365. 10.1016/j.intimp.2024.113365 39447408

[17] Bundkirchen K, Ye W, Zhang T et al (2025) A single acute alcohol intoxication before fracture insult causes long-term elevated systemic RANKL and OPG levels in young adult mice. Sci Rep 15:24423. 10.1038/s41598-025-09240-3 PMC12238551 40628859

[18] Komrakova M, Weidemann A, Dullin C et al (2015) The impact of strontium ranelate on metaphyseal bone healing in ovariectomized rats. Calcif Tissue Int 97:391–401. 10.1007/s00223-015-0019-0 26084691

[19] Tezval M, Stuermer EK, Sehmisch S et al (2010) Improvement of trochanteric bone quality in an osteoporosis model after short-term treatment with parathyroid hormone: a new mechanical test for trochanteric region of rat femur. Osteoporos Int 21:251–261. 10.1007/s00198-009-0941-y PMC2801842 19436940

[20] Sehmisch S, Erren M, Rack T et al (2009) Short-term effects of parathyroid hormone on rat lumbar vertebrae. Spine (Phila Pa 1976) 34:2014–2021. 10.1097/BRS.0b013e3181afe846 19730209

[21] Saul D, Gleitz S, Nguyen HH et al (2017) Effect of the lipoxygenase-inhibitors baicalein and zileuton on the vertebra in ovariectomized rats. Bone 101:134–144. 10.1016/j.bone.2017.04.011 28455215

[22] Komrakova M (2012) Efficiency of 48 h vs. 24 h injection of parathyroid hormone for amelioration of osteopenic spine properties in male rats. TOBONEJ 4:20–26. 10.2174/1876525401204010020

[23] Zhang T, Neunaber C, Ye W et al (2024) Aging influences fracture healing on the cellular level and alters systemic RANKL and OPG concentrations in a murine model. Adv Biol 8:2300653. 10.1002/adbi.202300653 39164219

[24] Zini G, Viscovo M (2021) Cytomorphology of normal, reactive, dysmorphic, and dysplastic megakaryocytes in bone marrow aspirates. Int J Lab Hematol 43(Suppl 1):23–28. 10.1111/ijlh.13536 34288439

[25] Bundkirchen K, Macke C, Angrisani N et al (2018) Hemorrhagic shock alters fracture callus composition and activates the IL6 and RANKL/OPG pathway in mice. J Trauma Acute Care Surg 85:359–366. 10.1097/TA.0000000000001952 29787542

[26] van ’t Hof RJ, Rose L, Bassonga E, Daroszewska A (2017) Open source software for semi-automated histomorphometry of bone resorption and formation parameters. Bone 99:69–79. 10.1016/j.bone.2017.03.051 28366796

[27] Tratwal J, Labella R, Bravenboer N et al (2020) Reporting guidelines, review of methodological standards, and challenges toward harmonization in bone marrow adiposity research. Rep Methodologies Working Group Int Bone Marrow Adiposity Soc Front Endocrinol 11:65. 10.3389/fendo.2020.00065 PMC7059536 32180758

[28] Prince RL, Lewis JR, Lim WH et al (2019) Adding lateral spine imaging for vertebral fractures to densitometric screening: improving ascertainment of patients at high risk of incident osteoporotic fractures. J Bone Min Res 34:282–289. 10.1002/jbmr.3595 30395687

[29] Khadka B, Tiwari ML, Gautam R et al (2018) Correlates of biochemical markers of bone turnover among post-menopausal women. JNMA J Nepal Med Assoc 56:754–758. 10.31729/jnma.3604 PMC8827533 30387463

[30] Greisen SR, Kragstrup TW, Thomsen JS et al (2022) The programmed death-1 pathway counter-regulates inflammation-induced osteoclast activity in clinical and experimental settings. Front Immunol 13:773946. 10.3389/fimmu.2022.773946 PMC8959817 35356000

[31] Schildberg FA, Haddouti E-M, Zhu M et al (2025) The PD-1 signalling pathway orchestrates bone homeostasis. Orthop Proc 107-B:4–4. 10.1302/1358-992X.2025.7.004

[32] Nagahama K, Aoki K, Nonaka K et al (2004) The deficiency of immunoregulatory receptor PD-1 causes mild osteopetrosis. Bone 35:1059–1068. 10.1016/j.bone.2004.06.018 15542030

[33] Smith L, Bigelow EMR, Jepsen KJ (2013) Systematic evaluation of skeletal mechanical function. Curr Protoc Mouse Biol 3:39–67. 10.1002/9780470942390.mo130027 PMC3948330 24634803

[34] Turner CH, Burr DB (1993) Basic biomechanical measurements of bone: a tutorial. Bone 14:595–608. 10.1016/8756-3282(93)90081-K 8274302

[35] Greisen SR, Kragstrup TW, Thomsen JS et al (2020) Programmed death ligand 2—a link between inflammation and bone loss in rheumatoid arthritis. J Transl Autoimmun 3:100028. 10.1016/j.jtauto.2019.100028 PMC7388353 32743513

[36] Gassner T, Chittilappilly C, Pirich T et al (2024) Favorable impact of PD1/PD-L1 antagonists on bone remodeling: an exploratory prospective clinical study and ex vivo validation. J Immunother Cancer 12:e008669. 10.1136/jitc-2023-008669 PMC11086513 38702145

[37] Lee S-C, Shin MK, Jang B-Y et al (2022) Immunomodulatory effect and bone homeostasis regulation in osteoblasts differentiated from hADMSCs via the PD-1/PD-L1 axis. Cells 11:3152. 10.3390/cells11193152 PMC9564087 36231113

[38] Brom VC, Strauss AC, Sieberath A et al (2023) Agonistic and antagonistic targeting of immune checkpoint molecules differentially regulate osteoclastogenesis. Front Immunol 14:988365. 10.3389/fimmu.2023.988365 PMC9931766 36817431

[39] Ye C, Lee K, Leslie WD et al (2023) Fracture rate increases after immune checkpoint inhibitor treatment: a potential new immune related adverse event. Osteoporos Int 34:735–740. 10.1007/s00198-023-06690-1 36729143

[40] Pantano F, Tramontana F, Iuliani M et al (2022) Changes in bone turnover markers in patients without bone metastases receiving immune checkpoint inhibitors: an exploratory analysis. J Bone Oncol 37:100459. 10.1016/j.jbo.2022.100459 PMC9633734 36338920

[41] Filippini DM, Gatti M, Di Martino V et al (2021) Bone fracture as a novel immune-related adverse event with immune checkpoint inhibitors: case series and large-scale pharmacovigilance analysis. Int J Cancer 149:675–683. 10.1002/ijc.33592 PMC8251715 33844854

[42] Wickham R (2011) Osteoporosis related to disease or therapy in patients with cancer. Clin J Oncol Nurs 15:E90–E104. 10.1188/11.CJON.E90-E104 22119987

[43] Costa SN, Chlebek C, Gray L et al (2026) Expansion of bone marrow adipocytes in obese mice leads to PD-L1-driven bone marrow immunosuppression and osteoclastogenesis. Bone Res 14:32. 10.1038/s41413-026-00509-5 PMC13002983 41856986

