# Supplementary material for: CD274 Knockout Promotes Bone Formation and Reduces Osteoclast Activity
Source: Calcif Tissue Int. 2026 Oct 20;117(1):164. doi: 10.1007/s00223-026-01627-2 (PMC13683541; doi:10.1007/s00223-026-01627-2)
Supplement: Supplementary file 1 [file 223_2026_1627_MOESM1_ESM.docx]

**Supplementary Information**

Article title: CD274 Knockout Promotes Bone Formation and Reduces Osteoclast Activity

Authors: Hadi El Assaad^1,2^, Marina Komrakova^3^, Stefan Lienenklaus^4^, Sebastian Decker^1^, Claudia Neunaber^1^, and Katrin Bundkirchen^1^

Affiliations:

1. Hannover Medical School, Department of Trauma Surgery, Carl-Neuberg-Straße 1, 30625 Hannover, Germany

2. Leipzig University Hospital, Department of Orthopedics, Trauma Surgery and Plastic Surgery, Liebigstraße 20, 04103 Leipzig, Germany

3. Goettingen University Medical Center, Department of Trauma Surgery, Orthopaedics and Plastic Surgery, Robert-Koch-Str. 40, 37075 Goettingen, Germany

4. Hannover Medical School, Institute of Laboratory Animal Science, Carl-Neuberg-Straße 1, 30625 Hannover, Germany

Corresponding author: Prof. Dr. Claudia Neunaber, neunaber.claudia@mh-hannover.de

**Supplementary Methods S1. Micro-CT image evaluation and region-of-interest (ROI) determination**

All micro-CT datasets were segmented in Inveon Research Workplace (Version 4.2, Siemens AG, Munich, Germany) on the transverse plane. For each sample, the materials “Total Bone”, “Trabecular Bone”, “Cortical Bone” and “Bone Marrow” were defined. Mineralised compartments were separated using fixed grey-value thresholds applied identically across all samples: total bone, 280 – maximum; cortical bone, 900 – maximum; trabecular bone, 280 – 900; and bone marrow, minimum – 280. Contours were drawn manually on every fifth transverse slice and interpolated across the intervening slices.

**Tibia.** The distal epiphyseal (trabecular) ROI began at the most distal slice containing tibial bone (adjacent to the ankle) and extended 125 slices proximally (≈ 1.02 mm). The diaphyseal (cortical) ROI began at the first slice proximal to the epiphysis and extended 600 slices (≈ 4.90 mm).

**Femur.** The distal epiphyseal (trabecular) ROI began at the most distal transverse slice containing femoral bone (adjacent to the knee) and extended 175 slices proximally (≈ 1.43 mm at an 8.17 µm effective voxel size). The diaphyseal (cortical) ROI began at the first slice proximal to the epiphysis and extended 600 slices (≈ 4.90 mm).

**Lumbar vertebra (L5).** The entire vertebral body was contoured from one endplate to the other, excluding the posterior elements.

Cortical wall thickness was measured at four positions per transverse section and averaged. From the segmented regions, volumetric parameters (total bone volume, cortical volume and trabecular volume) and morphometric parameters (cortical wall thickness, trabecular spacing, trabecular number, trabecular thickness and BV/TV) were calculated. Identical thresholds, material definitions and ROI conventions were applied to all animals in both groups. The corresponding regions are shown in Figure 1.

**Supplementary Methods S2. Quantification of marrow adiposity**

**Sections and stains**

Marrow adipocytes were quantified on the same 5 µm paraffin sections used for the other histological read-outs. The femur, humerus and lumbar vertebrae were assessed on Light Green/Safranin O-stained sections, in which the marrow is stained dark blue-grey, mineralised bone green and cartilage red, so that empty adipocyte profiles show the highest possible contrast. Digital images were acquired with a VHX 7000 digital microscope (Keyence GmbH, Germany) at 300x magnification.

**Regions of interest**

For the femur and humerus, the regions of interest had the size and shape of those used for the osteoclast counts (Table 1): one rectangle per bone, with its long axis along the shaft, centered in the medullary cavity and placed directly adjacent to the growth plate (femur 2.10 × 0.74 mm at the distal growth plate, humerus 1.16 × 0.57 mm at the proximal growth plate). For the lumbar vertebra, all marrow within one 300x field of the vertebral body (0.99 × 0.68 mm) was evaluated. The marrow area analysed per animal is given in Table S1.

**Table S1** Regions of interest used for marrow adiposity

| **Bone** | **Region of interest** | **Area (marrow area per animal)** |
| --- | --- | --- |
| Femur | one rectangle, 2.10 × 0.74 mm, at the distal growth plate | 1.55 mm^2^ (1.00 – 1.55 mm^2^) |
| Humerus | one rectangle, 1.16 × 0.57 mm, at the proximal growth plate | 0.66 mm^2^ (0.44 – 0.64 mm^2^) |
| Lumbar vertebra | one 300x field of the vertebral body, 0.99 × 0.68 mm | 0.67 mm^2^ (0.20 – 0.36 mm^2^) |

Marrow area (Ma.Ar) was defined as the cellular marrow area within the region of interest. Mineralised bone, cartilage, and torn or empty space were excluded, so that Ma.Ar reflects tissue that was actually present and could contain adipocytes.

**Identification criteria**

| **Counted as an adipocyte** | **Not counted** |
| --- | --- |
| Round to oval, sharply delimited, optically empty profile | Vessel lumina and sinusoids (contain erythrocytes, elongated or irregular) |
| Bordered by a thin, continuous membrane | Retraction clefts and tears, in particular crescent-shaped gaps along bone surfaces |
| Located within the marrow, surrounded by haematopoietic tissue | Empty osteocyte or chondrocyte lacunae |
| Equivalent diameter ≥ 12 µm | Megakaryocytes (large, but with stained cytoplasm and a lobulated nucleus) |
|  | Profiles in out-of-focus, torn or washed-out tissue |

**Detection and verification**

Detection and verification were separated into two steps so that neither an automated threshold nor the assessor had to be correct on its own.

1. Candidate profiles were detected over the whole section with a custom Python script (scikit-image, SciPy) using a deliberately over-inclusive rule: optically empty, low saturation, area 60 – 5000 µm², round, and surrounded by cellular marrow. The only purpose of this step is not to miss adipocytes.
2. Every candidate was then verified visually against the criteria above.

**Parameters**

- **N.Ad/Ma.Ar** – adipocyte number per marrow area (#/mm²)
- **Ad.Ar/Ma.Ar** – adipocyte area per marrow area (%)
- **Ad.Dm** – mean adipocyte diameter (µm), calculated per cell as the diameter of a circle of equal area and averaged over all adipocytes of one animal

**Supplementary Figure S1.** Representative Light Green/Safranin O staining. Humerus with boxes indicating the cortical regions of interest used for osteocyte counting (a); high-magnification detail of cartilage, which appears red adjacent to green-stained bone (b); high-magnification detail of cortical bone showing individual osteocytes as dark cells within the green-stained matrix (c) and a lumbar vertebra (d). Bone appears green and cartilage red. Osteocytes were counted within the boxed cortical regions; cartilage was quantified as the percentage of Safranin O–positive (red) area relative to total epiphyseal area. The dashed yellow outline in (a) marks the region in which marrow adipocytes were counted in the humerus (1.16 × 0.57 mm, 0.66 mm^2^, at the proximal growth plate).

**Supplementary Figure S2.** Representative TRAP staining used for osteoclast quantification, with regions of interest. (a) Femur, (b) proximal humerus and (c) lumbar vertebra; TRAP-positive osteoclasts appear red. Black outlines indicate the regions of interest in which osteoclasts were counted.

**Supplementary Figure S3.** Representative pentachrome staining of mineralized bone. (a) Femur, (b) humerus and (c) lumbar vertebra; mineralized bone appears yellow-orange. Mineralized bone was quantified as the percentage of yellow-orange area relative to the total area in the distal femur, proximal humerus (diaphysis and epiphysis) and vertebral body.

**Supplementary Figure S4.** Trabecular number and thickness in tibia and femur. Trabecular number (TbN, 1/mm) and trabecular thickness (TbTh, mm) of the tibia (a, b) and femur (c, d) in control and *Cd274* KO mice (n = 5–6/group), arranged to match Figure 2. Horizontal lines indicate the median and interquartile range. No significant differences were observed between groups.

**Supplementary Figure S5.** Representative histological sections of the distal femur, proximal humerus and lumbar vertebra 5 (L5) of a control (Ctrl) and a *Cd274* KO mouse. Light Green/Safranin O staining of cartilage in the femur (a, b) and humerus (c, d); cartilage appears red next to green-stained bone. Light Green/Safranin O staining used for the counting of osteocytes and marrow adipocytes in the femur (e, f), humerus (g, h) and L5 (i, j); osteocytes appear as dark cells within the green-stained matrix, in L5, all marrow within one 300x field of the vertebral body was evaluated for adipocytes count (i, j). TRAP staining in the femur (k, l), humerus (m, n) and L5 (o, p); TRAP-positive osteoclasts appear red. Pentachrome staining in the femur (q, r), humerus (s, t) and L5 (u, v); mineralized bone appears yellow-orange. Black outlines indicate the regions of interest used for counting. Arrows mark the cells or structures quantified and are shown only at the sites at which the difference between *Cd274* KO mice and controls was statistically significant: osteocytes in the humerus (g, h; p = 0.036) and L5 (i, j; p < 0.01), osteoclasts in the humerus (m, n; p = 0.044) and L5 (o, p; p = 0.002), and mineralized bone in L5 (u, v; p = 0.014). No arrows are shown where the difference was not significant.
